# Mechanistic insights into vascular biology via methyltransferase-like 3-driven N^6^-adenosine methylation of RNA

**DOI:** 10.3389/fcell.2024.1482753

**Published:** 2025-01-06

**Authors:** Deshuang Zhang, Zhixian Gou, Yi Qu, Xiaojuan Su

**Affiliations:** ^1^ Department of Pediatrics, Key Laboratory of Birth Defects and Related Diseases of Women and Children (Ministry of Education), NHC Key Laboratory of Chronobiology, West China Second University Hospital, Sichuan University, Chengdu, Sichuan, China; ^2^ Division of Neonatology, Department of Pediatrics, The Affiliated Hospital of Southwest Medical University, Luzhou, Sichuan, China; ^3^ Department of Pediatrics, School of Clinical Medicine and The First Affiliated Hospital of Chengdu Medical College, Chengdu, China

**Keywords:** methyltransferase-like 3, pericytes, endothelial cells, vascular smooth muscle cells, hematopoietic stem cells, vascular biology

## Abstract

Recent advancements in the mechanistic comprehension of vascular biology have concentrated on METTL3-mediated N^6^-methyladenosine modification of RNA, which modulates a spectrum of RNA functionalities with precision. Despite extensive investigations into the roles and mechanisms of METTL3 within vascular biology, a holistic review elucidating their interconnections remains absent. This analysis endeavors to meticulously scrutinize the involvement of METTL3 in both the physiological and pathological paradigms of vascular biology. The findings of this review indicate that METTL3 is indispensable for vascular development and functionality, predominantly through its regulatory influence on pericytes, endothelial cells, vascular smooth muscle cells, and hematopoietic stem cells. Conversely, aberrant METTL3 activity is implicated as a risk factor, diagnostic biomarker, and therapeutic target for vascular pathologies. This comprehensive review offers an exhaustive synthesis of METTL3’s role in vascular biology, addressing existing knowledge gaps and serving as an essential reference for future research and potential clinical applications.

## Graphical Abstract

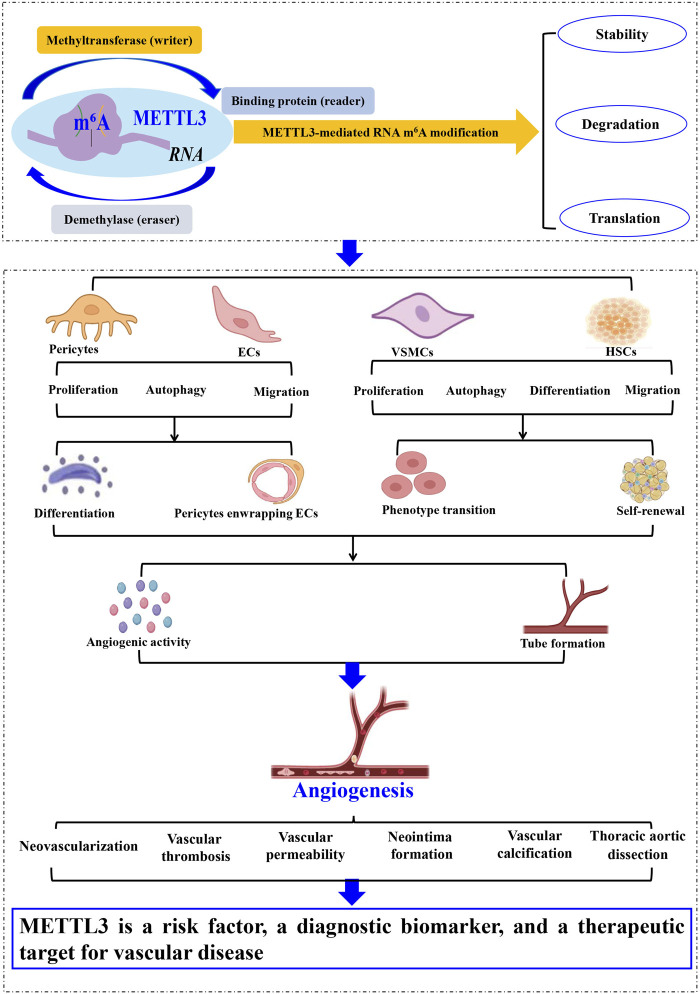

## Highlights

1. METTL3 sustains optimal angiogenic processes and vascular functionality
2. METTL3 plays a pivotal role in pericyte-mediated microvascular pathology
3. METTL3 is essential for preserving EC functions during angiogenesis
4. METTL3 regulates VSMC functions in angiogenic activity and vascular calcification
5. METTL3 governs the self-renewal and phenotypic transition of HSCs

## 1 Introduction

The ontogenesis and functionality of the vasculature are complex and crucial for the systemic distribution of various biomolecules (Liu et al., 2024). Angiogenesis, the formation of new blood vessels from pre-existing vasculature, primarily involves cellular proliferation, migration, and differentiation (Liu et al., 2024). The functionality of vascular structures depends on an intricate cellular network that maintains the homeostasis of arteries, capillaries, and veins (Liu et al., 2024). Proper regulation of cellular biological functions is essential for sustaining optimal angiogenesis and vascular operations.

The mechanistic understanding of cellular function regulation has attracted significant interest within the epigenetic domain, particularly focusing on the prevalent N^6^-methyladenosine (m^6^A) RNA modification. The m^6^A modification governs mRNA splicing, localization, translation, and stability through a coordinated process mediated by methyltransferases (writers), demethylases (erasers), and binding proteins (readers) (Wang et al., 2020). The methyltransferase complex, primarily composed of methyltransferase-like 3/14 (METTL3/14) and Wilms tumor 1-associated protein (WTAP), is pivotal in preserving the m^6^A modification state (Petri and Klinge, 2023). Conversely, m^6^A demethylation is facilitated by demethylases, mainly comprising alkylation repair homolog protein 5 (ALKBH5) and fat mass and obesity-associated protein (FTO) (Petri and Klinge, 2023). Readers, such as YTH N^6^-methyladenosine RNA binding proteins (YTHDF) and insulin-like growth factor 2 mRNA binding proteins (IGF2BP), recognize and interpret m^6^A marks on RNAs (Petri and Klinge, 2023). The interplay among these m^6^A components (writers, erasers, and readers) orchestrates gene functions like mRNA stability, translation, and degradation, thereby determining cellular outcomes such as proliferation, phenotype, migration, and differentiation (Petri and Klinge, 2023).

METTL3 stands as the sole catalytically active regulator within the m^6^A RNA modification process (Su et al., 2024). Contemporary insights into cell function regulation via METTL3-mediated m^6^A modification have enhanced our mechanistic comprehension of vascular health and pathology. Despite extensive research on METTL3’s roles and mechanisms in vascular biology, there are limited reviews elucidating their interrelationship and assessing METTL3’s potential clinical applications.

This review aims to systematically analyze extant literature regarding METTL3’s involvement in the physiological and pathological paradigms of vascular biology. Furthermore, we appraise METTL3’s viability as a clinical target for diagnosing and treating vascular diseases. Collectively, this review illuminates the nexus between METTL3-mediated RNA m^6^A regulation and vascular biology, bridging knowledge gaps and serving as a valuable reference for foundational research and prospective clinical applications.

## 2 Correlation between METTL3 and vascular physiology and pathology

Vascular phenomena encompass vasculogenesis, angiogenesis, and associated pathologies. The vasculogenic process encompasses multiple phases, predominantly involving the functions and interactions of pericytes and endothelial cells (ECs), which are spatially proximate within the capillaries (Su et al., 2019). Specifically, neovascular sprouting marks the commencement of angiogenesis, during which pericytes facilitate EC maturation and secrete vascular endothelial growth factor (VEGF) to initiate new vessel formation (Su et al., 2019). Subsequently, pericytes secrete VEGF and fibroblast growth factor (FGF) to enhance EC migration, proliferation, aggregation, and differentiation, ultimately contributing to the stabilization and extension of nascent blood vessels. Following this, pericytes interact with ECs to integrate the newly formed vasculature. Finally, pericytes synthesize the extracellular matrix (ECM) to fortify the nascent vessels. Beyond their role in vasculogenesis, pericytes and ECs, in concert with vascular smooth muscle cells (VSMCs) and hematopoietic stem cells (HSCs), are pivotal in maintaining vascular functionality, with any dysfunction precipitating vascular pathologies.

METTL3 has been implicated in the regulation of angiogenesis and vascular functionality, primarily through its role in modulating the biological activities of pericytes, ECs, VSMCs, and HSCs.

### 2.1 METTL3 modulates pericyte physiology and pathology in vascular

**FIGURE 1:**
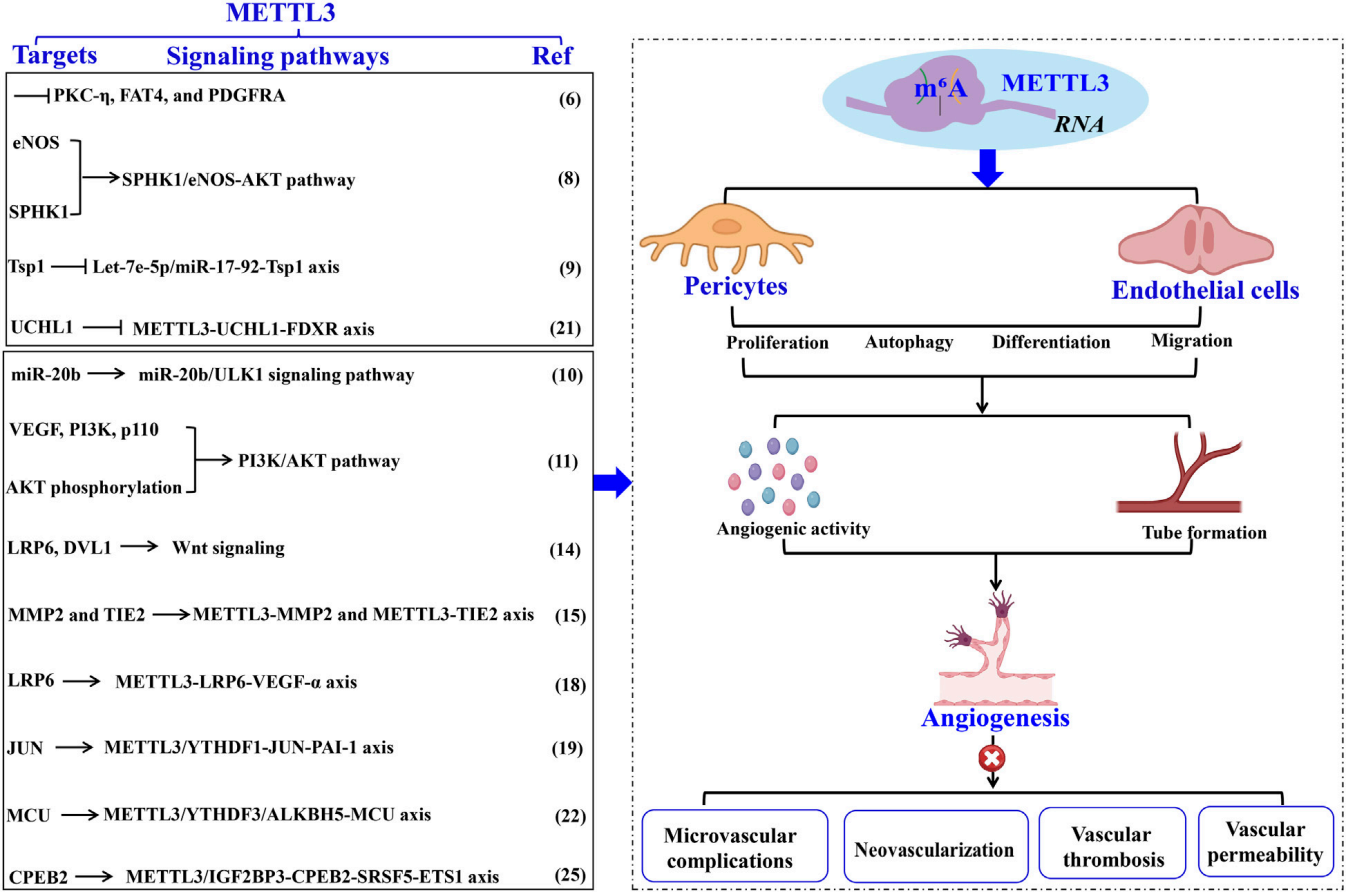
The role of METTL3 in vascular pathology through EC modulation. METTL3-driven RNA m^6^A modification intricately controls EC functions, thereby facilitating aberrant angiogenesis and vascular pathologies. The specific targets, underlying mechanisms, and the impact of METTL3 in vascular diseases are comprehensively delineated. Methyltransferase-like 3 (METTL3). Α-smooth muscle actin (α-SMA). Endothelial cell (EC). N^6^-methyladenosine (m^6^A). Endothelial nitric oxide synthase (eNOS). Phosphorylation and sphingosine kinase 1 (SPHK1). Thrombospondin 1 (TSP1). Ferredoxin reductase (FDXR). Unc-51- like kinase 1 (ULK1). Vascular endothelial growth factor (VEGF). Matrix metalloproteinase (MMP). LDL receptor-related protein 6 (LRP6). Disheveled 1 (DVL1). N^6^-Methyladenosine RNA binding protein F (YTHDF). Plasminogen activator inhibitor-1 (PAI-1). Mitochondrial calcium uniporter (MCU). Alkylation repair homolog protein 5 (ALKBH5).

Pericytes, specialized contractile cells located along capillary walls, extensively envelop capillaries, arterioles, and venules throughout the body. As critical constituents of the vasculature, pericytes exist in various subtypes and primarily exert their functions through the secretion of distinct signaling molecules. For instance, pericytes modulate vascular tone and blood flow by contracting and expressing α-smooth muscle actin (α-SMA), thereby maintaining tissue homeostasis and metabolic equilibrium (Su et al., 2019). Nonetheless, pericyte dysfunction is implicated in microvascular complications, closely linked to METTL3-mediated RNA m^6^A modifications. Suo et al. (2022) demonstrated that METTL3 is markedly upregulated in pericyte dysfunction induced by diabetic stress, adversely affecting pericyte viability, proliferation, and differentiation by inhibiting the expression of PKC-η, FAT4, and PDGFRA in a YTHDF2-dependent mechanism. These observations underscore the critical role of METTL3 in sustaining pericyte functionality, while its dysregulation precipitates pericyte malfunction. Given the paucity of research on METTL3’s regulatory role in pericytes, further investigations are warranted to delineate its regulatory network and potential clinical applications (Figure 1; Table 1).

**TABLE 1:** Functions and targets of METTL3 in vascular biology.

METTL3				
Targets	Functions	Cells	Events	Ref
*PKC-η*, *FAT4, PDGFRA*	Inhibits the expression of PKC-η, FAT4, and PDGFRA in an YTHDF2-dependent mechanism	Pericytes	Diabetic stress	Suo et al. (2022)
*eNOS, SPHK1*	Modulates eNOS phosphorylation and SPHK1 expression with ALKBH5	ECs	Abnormal Angiogenesis	Kumari et al. (2021)
*let-7e-5p, miR-17–92*	Enhances levels of mature let-7e-5p and miR-17-92	ECs	Abnormal Angiogenesis	Chamorro-Jorganes et al. (2021)
*miR-20b*	Enhances miR-20b expression	ECs	Abnormal Angiogenesis	Lu et al. (2020)
*VEGF, PI3K p110, AKT*	Increases VEGF and PI3K p110 levels and AKT phosphorylation	EPCs	Hypoxia-induced angiogenesis and distraction osteogenesis	Jiang et al. (2021)
*LRP6, DVL1*	Enhances the translation of LRP6 and DVL1 in an YTHDF1-dependent manner	RECs	Burn-induced corneal neovascularization	Yao et al. (2020)
*MMP2, TIE2*	Facilitates m^6^A modification on MMP2 and TIE2 to enhance their expression	HRMEC	Oxygen-induced retinopathy	Lin et al. (2023)
*LRP6*	Enhances LRP6 stability	HUVEC	Corneal neovascularization	Wang et al. (2023)
*JUN*	Enhances the translation of JUN mRNA in an YTHDF1 manner	HUVEC	Endotoxin-induced thrombosis	Bai et al. (2021)
*UCHL1*	Decreases UCHL1 expression via m^6^A modification-mediated mRNA decay and R-loop-dependent transcriptional termination	EC	HCMV-induced AS	Zhu et al. (2024)
*MCU*	Facilitates YTHDF3 interaction and enhances MCU translation and expression	EC	HCMV-induced AS	Zhu et al. (2022)
*CPEB2*	Augments the stability of CPEB2 mRNA via m^6^A methylation	Glioma microvascular EC	Blood-tumor barrier permeability	Zhang et al. (2022)
*a-SMA, sm22α, VEGF, HGF, TGF-β, FGF, SDF-1*	Suppresses the expression of a-SMA, sm22α, and calponin. Augments the secretion of VEGF, HGF, TGF-β, FGF, and SDF-1	VSMC	Angiogenesis	Lin et al. (2020)
*p-mTOR, p-CDK1, CDC2*	Decreases p-mTOR and p-CDK1/CDC2 levels	VSMC	Neointima Formation	Luo et al. (2023)
*ATG5* *ATG7*	Downregulates ATG5 and ATG7	HASMC	Neointima formation	Fang et al. (2023)
*PI3K*	Reduces PI3K mRNA decay	VSMC	Neointimal hyperplasia	Zhao et al. (2022)
*NOTCH1*	Enhances m^6^A modification on NOTCH1 mRNA and facilitates NOTCH1 mRNA degradation via collaboration with YTHDF2	HASMC	Thoracic aortic dissection	Yang et al. (2023)
*rip3*	Enhances the m^6^A modification of rip3 mRNA by increases the binding of YTHDF3 to rip3 mRNA	SMC	Abdominal aortic aneurysm	Ji et al. (2023)
*microRNA-34a*	Facilitates the maturation of primary microRNA-34a into miR-34a by targeting DGCR8			Li et al. (2022)
*Myc*	Enhances the abundance of Myc mRNA	HSC	Angiogenesis	Lee et al. (2019), Cheng et al. (2019)
*Notch1*	PromotesYTHDF2-mediated decay of Notch1 mRNA	HSC	Embryo development	Lv et al. (2018)
*Notch1a* *Rhoca*	Promotes YTHDF2-mediated mRNA decay of Notch1a and Rhoca	HSC	Obstructs embryo development	Zhang et al. (2017)

Methyltransferase-like 3 (METTL3). Endothelial cell (EC). N^6^-methyladenosine (m^6^A). Endothelial nitric oxide synthase (eNOS). Phosphorylation and sphingosine kinase 1 (SPHK1). Thrombospondin 1 (TSP1). Ferredoxin reductase (FDXR). Unc-51- like kinase 1 (ULK1). Vascular endothelial growth factor (VEGF). Matrix metalloproteinase (MMP). LDL, receptor-related protein 6 (LRP6). Disheveled 1 (DVL1). Plasminogen activator inhibitor-1 (PAI-1). Mitochondrial calcium uniporter (MCU). Alkylation repair homolog protein 5 (ALKBH5). Α-smooth muscle actin (α-SMA). Smooth muscle 22α (sm22α). Vascular smooth muscle cells (VSMC). Smooth muscle cells (SMCs). Hepatocyte growth factor (HGF). Hematopoietic stem cells (HSC). Cyclin-dependent kinase 1 (CDK1). Autophagy-related 5 (ATG). N^6^-methyladenosine RNA, binding protein F (YTHDF).

### 2.2 METTL3 modulates EC functions in vascular physiology and pathology

ECs constitute the innermost lining of the vascular endothelium, playing crucial roles in neovascularization (Jeong et al., 2021). Newly formed vessels integrate into the vascular endothelium to perform their functions, thereby highlighting the importance of ECs in vessel formation and functionality.

#### 2.2.1 METTL3’s role in regulating angiogenesis

Extensive research has underscored the pivotal role of METTL3 in modulating the biological functions of ECs implicated in angiogenesis. Kumari et al. (2021) demonstrated that elevated ALKBH5 levels in ECs facilitate sustained angiogenesis post-acute ischemia. ALKBH5 modulates endothelial nitric oxide synthase (eNOS) phosphorylation and sphingosine kinase 1 (SPHK1) expression through METTL3-mediated m^6^A methylation. These findings imply that increased ALKBH5 expression orchestrates EC functions in angiogenesis via the METTL3/SPHK1/eNOS-AKT signaling cascade, underscoring METTL3’s promotive role in angiogenesis. Corroborating this, another study revealed that METTL3 knockdown in ECs hampers angiogenesis (Chamorro-Jorganes et al., 2021). The suppression of METTL3 in ECs results in reduced levels of mature angiogenic microRNAs, such as let-7e-5p and the miR-17-92 cluster, while concurrently upregulating their shared target, thrombospondin 1 (TSP1), thereby impairing the angiogenic capacity of ECs (Chamorro-Jorganes et al., 2021). Conversely, METTL3 overexpression or augmentation of let-7e-5p and miR-18a-5p levels effectively reinstates EC angiogenic functionality. Collectively, these observations suggest that a sustained high level of METTL3 is critical for optimal EC function in angiogenesis, potentially through modulation of the let-7e-5p/miR-17-92-TSP1 axis.

Additionally, maintaining elevated METTL3 levels is vital for addressing angiogenic disorders. Lu et al. (2020) found that *Propofol* upregulates miR-20b and METTL3 expression during hypoxia/re-oxygenation-induced EC autophagic death, thereby mitigating cellular damage. METTL3 depletion leads to diminished miR-20b expression. Inhibition of miR-20b or METTL3 attenuates *Propofol’s* protective effect, exacerbating autophagy in ECs by targeting Unc-51-like kinase 1 (ULK1). These results indicate that *Propofol* confers protection against EC death via the METTL3/miR-20b-ULK1 signaling pathway. Furthermore, Jiang et al. (2021) reported a significant upregulation of METTL3 in endothelial progenitor cells (EPCs) during hypoxia-induced angiogenesis and distraction osteogenesis (DO), enhancing EC proliferation, tube formation, migration, and angiogenic activity. METTL3 upregulation promotes bone regeneration during DO by increasing VEGF and PI3K p110 levels and AKT phosphorylation, thereby facilitating EPC-induced angiogenesis through the PI3K/AKT pathway (Jiang et al., 2021). These insights propose that METTL3 overexpression is a promising therapeutic approach to augment bone regeneration in DO (Figure 1; Table 1).

#### 2.2.2 METTL3 modulates EC activity in neovascular processes

Retinal neovascularization is a critical feature of various ocular pathologies, such as retinopathy of prematurity, diabetic retinopathy, and retinal vein occlusion (Heath Jeffery and Chen, 2024). The primary etiologies of this aberrant angiogenic response encompass ischemic hypoxia, inflammatory processes, and viral infections within the retina, leading to proteolytic degradation of the basement membrane maintained by ECs (Heath Jeffery and Chen, 2024). The resultant loss of structural support precipitates increased EC migration and proliferation, culminating in angiogenesis and neovascular formations.

Retinal ischemia and hypoxia precipitate in neovascularization, which is closely associated with the upregulation of several angiogenesis-related factors that modulate retinal endothelial cell (REC) proliferation, migration, and angiogenesis, including VEGF, erythropoietin, and angiopoietins (Fevereiro-Martins et al., 2023). Under hypoxic conditions, Yao et al. (2020) identified a significant upregulation of METTL3 expression in both ECs and murine retinas, which subsequently promotes pathological angiogenesis. Mechanistically, the increased expression of METTL3 synergizes with YTHDF1 to enhance the translation of low-density lipoprotein (LDL) receptor-related protein 6 (*LRP6*) and disheveled 1 (*DVL1*) via Wnt signaling modulation, thereby promoting heightened EC viability, proliferation, migration, and aberrant tube formation. Conversely, METTL3 ablation in a hypoxia-induced retinopathy model results in reduced avascular regions, diminished pathological neovascular tufts, and inhibition of alkali burn-induced corneal neovascularization (CNV) (Yao et al., 2020). These findings imply that METTL3-mediated regulation of LRP6/DVL1-Wnt signaling under hypoxic stress may be a pivotal mechanism in modulating normal angiogenesis. Consistently, Lin et al. (2023) revealed that both METTL3 expression and m^6^A modification levels are significantly elevated in human retinal microvascular endothelial cells (HRMECs), as well as in retinas and RECs under hypoxic conditions. The elevated METTL3 levels facilitate m^6^A modification on matrix metalloproteinase 2 (*MMP2*) and *TIE2*, enhancing their protein expression, which in turn augments endothelial tube formation and migration (Lin et al., 2023). Notably, the knockdown of MMP2 and TIE2 significantly impairs the angiogenic capabilities of HRMECs. Collectively, these findings indicate that METTL3-mediated m^6^A modifications enhance EC angiogenic behaviors by targeting *MMP2* and *TIE2*, underscoring the importance of the METTL3-MMP2 and METTL3-TIE2 axes in retinal angiogenesis and positioning METTL3 as a potential therapeutic target for oxygen-induced retinopathy.

Furthermore, herpes stromal keratitis (HSK) is a potentially vision-impairing condition caused by herpes simplex virus type 1 (HSV-1) infection (Stepp and Menko, 2021). CNV is a clinical manifestation of HSK, leading to irreversible visual impairment and blindness. Although HSV-1 does not encode pro-angiogenic proteins at any infection stage, it induces the production of various growth factors that result in CNV, with VEGF-α playing a predominant role. CNV induced by HSV-1 is significantly influenced by VEGF-α, and some studies suggest that virus-infected ECs are a major source of VEGF-α during early HSV-1 infection (Petti and Lodi, 2019). Therefore, inhibiting VEGF-α in ECs substantially mitigates the progression of HSK. Given the critical role of METTL3 in EC angiogenic properties, the mechanism underlying VEGF-α inhibition in ECs might involve METTL3. Wang et al. (2023) demonstrated that METTL3 is markedly upregulated in HSV-1-infected human umbilical vein endothelial cells (HUVECs), leading to increased m^6^A levels. Mechanistically, METTL3 regulates LRP6 expression through m^6^A-dependent post-transcriptional mRNA modification, enhancing its stability, upregulating VEGF-α expression, and promoting angiogenesis in HSV-1-infected HUVECs. Moreover, METTL3 knockdown or inhibition via STM2457 further reduces m^6^A levels and VEGF-α expression, impairs HUVEC migration and tube formation, and diminishes CNV *in vivo* following HSV-1 infection. Thus, METTL3 facilitates pathological angiogenesis through canonical Wnt and VEGF signaling pathways both *in vitro* and *in vivo*, highlighting METTL3 as a potential pharmacological target for preventing CNV progression in HSK (Figure 1; Table 1).

#### 2.2.3 METTL3 modulates EC functions in vascular thrombosis

Bai et al. (2021) observed a marked upregulation of METTL3 in the vasculature of mice and HUVECs subjected to lipopolysaccharide-induced inflammatory insult. This upregulation synergizes with YTHDF1 to enhance the translation of *JUN* mRNA, thereby elevating JUN expression. Consequently, JUN binds to the promoter of plasminogen activator inhibitor-1 (PAI-1), augmenting PAI-1 expression and promoting fibrin deposition in endotoxin-induced thrombosis. Conversely, EC-specific deletion of METTL3 diminishes PAI-1 plasma levels and facilitates fibrinolysis in endotoxin-induced thrombosis in mice. Thus, METTL3 exacerbates EC-mediated coagulation and thrombosis through the regulation of the JUN-PAI-1 axis, suggesting METTL3 as a promising therapeutic target for endotoxin-induced thrombosis.

Atherosclerosis (AS) is the predominant form of thrombosis (Patti et al., 2023). Human cytomegalovirus (HCMV) infection is a likely etiological factor for AS and restenosis post-vascular transplantation, as it induces inflammatory alterations in the vascular endothelium (Patti et al., 2023). Zhu et al. (2024) revealed that HCMV infection involves METTL3-mediated m^6^A modification in ECs. Specifically, HCMV infection triggers abnormal upregulation of m^6^A modification and R-loops in ECs, while decreasing UCHL1 expression via m^6^A modification-mediated mRNA decay and R-loop-dependent transcriptional termination. UCHL1 deficiency leads to the ubiquitination and degradation of ferredoxin reductase (FDXR), resulting in mitochondrial iron overload and AIM2 inflammasome activation, culminating in endothelial injury. Notably, METTL3 inhibition via the specific inhibitor STM2457 restores UCHL1 expression during HCMV infection, mitigating inflammatory injury in vascular ECs. These findings delineate the METTL3-UCHL1-FDXR axis as a novel mechanism for HCMV-induced endothelial injury, indicating the therapeutic potential of METTL3 suppression in HCMV-induced AS. Moreover, HCMV infection induces apoptosis in vascular ECs, further contributing to AS progression (Zhu et al., 2022). HCMV infection upregulates METTL3 and YTHDF3 expression, with METTL3 methylating mitochondrial calcium uniporter (*MCU*) mRNA, facilitating YTHDF3 interaction and enhancing MCU translation and expression. In contrast, ALKBH5 negatively regulates *MCU* mRNA by demethylation. This study highlights that the METTL3/YTHDF3/ALKBH5 axis governs endothelial cell apoptosis by targeting *MCU*. Collectively, these insights suggest METTL3 suppression as a therapeutic approach for HCMV-induced AS. For instance, Zhu et al. (2022) demonstrated that vitamin D3 protects ECs from HCMV-induced apoptosis by inhibiting both endoplasmic reticulum and mitochondrial apoptosis pathways. Vitamin D3 downregulates METTL3 expression by inactivating the AMPK pathway, thereby reducing m^6^A modification on MCU and preventing cell apoptosis. These findings expand our understanding of virus-induced endothelial damage mechanisms and underscore the therapeutic significance of vitamin D3 in HCMV-induced AS (Figure 1; Table 1).

#### 2.2.4 METTL3 modulates EC functions in vascular permeability

The blood-tumor barrier (BTB) poses a significant challenge to therapeutic efficacy by restricting drug penetration; thus, enhancing BTB permeability is crucial for glioma treatment (Ponmozhi et al., 2023). Tight junctions, constituted by tight-junction-associated proteins (TJPs), are pivotal targets for modulating BTB permeability (Scalise et al., 2021). Zhang et al. (2022) demonstrated that METTL3-driven m^6^A modification is instrumental in regulating BTB permeability. Specifically, glioma microvascular ECs exhibit aberrant upregulation of METTL3 and IGF2BP3, which augments the stability of *CPEB2* mRNA via m^6^A methylation. CPEB2 subsequently interacts with and stabilizes *SRSF5* mRNA, which facilitates the SRSF5-mediated alternative splicing of *ETS1*. Consequently, the METTL3/IGF2BP3- CPEB2-SRSF5-ETS1 pathway upregulates TJPs, including zonula occludens-1 (ZO-1), occludin, and claudin-5, thereby reducing BTB permeability. *In vivo* silencing of METTL3 or CPEB2 in glioblastoma xenograft models results in diminished expression of ZO-1, occludin, and claudin-5, leading to increased BTB permeability and enhanced glioma-targeted chemotherapeutic efficacy. Thus, METTL3 is a key regulator of BTB permeability, offering insights into glioma treatment strategies targeting METTL3 (Figure 1; Table 1).

In summary, sustaining elevated levels of METTL3 under physiological conditions is critical for EC function in angiogenesis; whereas under stress response conditions, METTL3 exacerbates pathological features. Therefore, we propose that METTL3 serves as a potential diagnostic biomarker and therapeutic target for EC dysfunction-related vascular disorders.

### 2.3 METTL3 modulates VSMC functions in vascular physiology and pathology

VSMCs exhibit significant plasticity in their regulation of vascular biology, oscillating between a dedifferentiated (synthetic, proliferative) state and a differentiated (contractile) phenotype by modulating the expression of various genes and proteins. In particular, following vascular injury, VSMCs adopt a highly dedifferentiated phenotype, marked by substantial proliferation, migration, and reduced expression of contractile-associated genes such as a-SMA and smooth muscle protein 22-alpha (sm22α), which eventually contribute to VSMC-driven vascular diseases like neointimal hyperplasia, calcification, and AS (Frismantiene et al., 2018).

#### 2.3.1 METTL3 governs the phenotypic transition of VSMCs in angiogenesis

**FIGURE 2:**
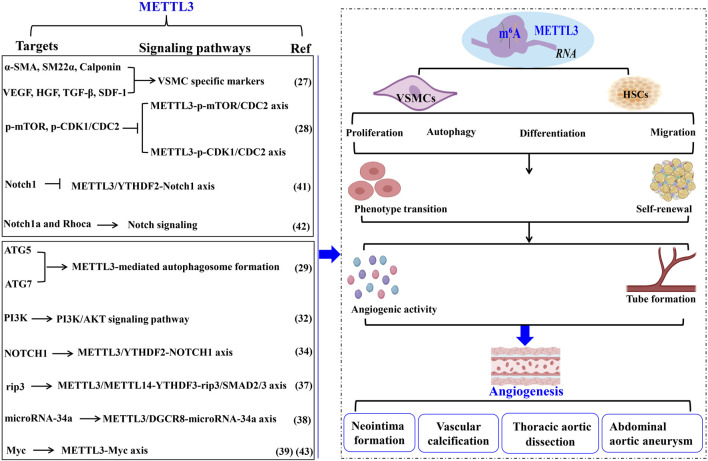
METTL3 modulates vascular pathophysiology through VSMC regulation. The biological activities of VSMCs in pathological angiogenesis and vascular disorders are modulated by METTL3 and its RNA m^6^A methylation activity. This figure delineates the targets, underlying mechanisms, and functional roles of METTL3 in these vascular processes. Methyltransferase-like 3 (METTL3). N^6^-methyladenosine (m^6^A). Α-smooth muscle actin (α-SMA). Smooth muscle 22α (sm22α). Vascular smooth muscle cells (VSMC). Hepatocyte growth factor (HGF). Cyclin-dependent kinase 1 (CDK1). Autophagy-related 5 (ATG). N^6^-Methyladenosine RNA binding protein F (YTHDF). Smooth muscle cells (SMCs). Abdominal aortic aneurysm (AAA). Extracellular matrix (ECM). Smooth muscle cells (SMCs). Hematopoietic stem cells (HSCs).

The phenotypic transition of VSMCs has been linked to METTL3 activity. Lin et al. (2020) demonstrated that hypoxic conditions markedly enhance METTL3 expression in VSMCs. Elevated METTL3 levels facilitate VSMC proliferation and generation while suppressing the expression of a-SMA, sm22α, calponin, and smooth muscle myosin heavy chain. Furthermore, METTL3 overexpression induces the differentiation of adipose-derived stem cells into VSMCs and augments the secretion of angiogenesis-promoting factors, including VEGF, HGF, TGF-β, FGF, and SDF-1, collectively enhancing VSMC-driven angiogenesis (Figure 2; Table 1).

#### 2.3.2 METTL3 regulates VSMC activities in neointima formation

While VSMC proliferation is essential for maintaining vascular architecture, pathological conditions result in both excessive and insufficient VSMC proliferation, adversely affecting the organism. METTL3 significantly influences VSMC proliferation, contributing to neointima formation post-vascular injury. Luo et al. (2023) found that METTL3 modulates VSMC autophagy to promote neointima formation by decreasing p-mTOR and cyclin-dependent kinase 1 (p-CDK1/CDC2) levels, thereby inhibiting VSMC proliferation through autophagosome formation. Similarly, Fang et al. (2023) indicated that METTL3 downregulation during VSMC proliferation and neointima formation suppresses VSMC phenotypic switching by activating autophagosome formation. METTL3 enhances autophagosome formation by upregulating autophagy-related genes *ATG5* and *ATG7*. Knockdown of ATG5 or ATG7 substantially reverses METTL3’s regulatory impact on human aortic smooth muscle cells (HASMCs) phenotypic switching. METTL3 knockdown promotes, while its overexpression suppresses, HASMC proliferation by arresting cells at the G2/M checkpoint and deactivating CDC2 phosphorylation (p-CDC2). Notably, aberrant VSMC proliferation and autophagosome formation contribute to neointima formation, a primary cause of poor outcomes post-stenting or coronary artery bypass grafting, regulated by METTL3. Additionally, METTL3 knockdown enhances HASMC migration and synthetic phenotype, whereas its overexpression inhibits these processes. Overexpression of METTL3 reduces MMP2, MMP7, and MMP9 protein levels while increasing tissue inhibitor of metalloproteinase 3 expression in HASMCs. These findings suggest that METTL3 impedes VSMC phenotypic switching by positively regulating ATG5 and ATG7-mediated autophagosome formation. Thus, mitigating neointima formation necessitates preventing abnormal VSMC proliferation and autophagosome formation by targeting METTL3, thereby improving long-term outcomes following stenting or coronary artery bypass grafting (Figure 2; Table 1).

#### 2.3.3 METTL3 modulates VSMC functionalities in AS pathogenesis

Moreover, the phenotypic shift of VSMCs from a differentiated to a dedifferentiated state is pivotal in AS pathogenesis (Zhai et al., 2022). Vascular calcification stands as a clinical hallmark of AS. Han et al. (2021) elucidated that METTL3 expression is markedly elevated in VSMCs during vascular calcification instigated by oxidized LDL, acting as a target for miR-33a-5p. Similarly, Zhao et al. (2022) established that METTL3 is a crucial agent in catalyzing a global increase in m^6^A methylation in response to carotid artery injury and diverse VSMC phenotype transitions. Elevated METTL3 expression induces a global rise in m^6^A modifications, thereby augmenting VSMC proliferation and migration while suppressing contractile gene expression. Conversely, *Mettl3* ablation mitigates this phenotypic transition in VSMCs, evidenced by reduced m^6^A levels, diminished proliferation, migration, neointimal hyperplasia, and upregulated contractile gene expression (Zhao et al., 2022). Mechanistically, *Mettl3* silencing enhances *PI3K* mRNA decay, thereby deactivating the PI3K/AKT signaling pathway to inhibit VSMC phenotypic switching. Collectively, these results underscore the significance of METTL3-mediated m^6^A in VSMC phenotypic modulation, proposing METTL3 as a therapeutic target for AS and restenosis (Figure 2; Table 1).

#### 2.3.4 METTL3 governs VSMC functions in thoracic aortic dissection (TAD)

The phenotypic transition of HASMCs similarly contributes to TAD, characterized by dedifferentiated VSMCs (Chakraborty et al., 2023). Yang et al. (2023) discovered that METTL3 expression is significantly elevated in HASMCs, fostering extensive HASMC proliferation by repressing α-SMA, sm22α, calponin, and NOTCH1. Mechanistically, METTL3 enhances m^6^A modification on *NOTCH1* mRNA and facilitates *NOTCH1* mRNA degradation via collaboration with YTHDF2, thereby inducing HASMC phenotypic alteration. METTL3 knockdown exhibits contrary effects. These findings indicate that METTL3 orchestrates HASMC phenotypic changes by upregulating m^6^A modifications on *NOTCH1* and suppressing NOTCH1 expression, consequently driving TAD development. In summary, elevated METTL3 levels promote VSMC formation and angiogenesis, playing a critical role in TAD (Figure 2; Table 1).

#### 2.3.5 METTL3 regulates VSMC activity in abdominal aortic aneurysm (AAA)

AAA, or abdominal aortic aneurysm, is characterized by a localized abnormal dilation of blood vessels, often resulting from the thinning of arterial walls due to vascular injury (Li et al., 2023). Key histopathological features of AAA include chronic inflammation of the vascular wall, apoptosis of smooth muscle cells (SMCs), and remodeling of the extracellular matrix (ECM) (Li et al., 2023). SMCs are the predominant cell type in the middle layer of the aortic wall (Zhong et al., 2020). An imbalance between SMC proliferation and apoptosis is pivotal in the remodeling of the aortic wall and the progression of AAA (Chakraborty et al., 2023). In the context of angiotensin II (Ang II)-induced AAA, there is an observed increase in necroptosis and inflammatory cytokines within vascular SMCs (Ji et al., 2023). Additionally, levels of m6A modification and the expression of METTL3 and METTL14 are heightened in the aortic wall tissues affected by AAA, indicating a significant association with Ang II-induced aneurysm formation (Ji et al., 2023). Ji et al. (2023) demonstrated that the METTL3-METTL14 complex interacts with rip3 mRNA and SMAD2/3 through YTHDF3 in the vascular SMCs of Ang II-induced AAA. Ang II activation stimulates SMAD2/3 in the abdominal aortic wall SMCs, which subsequently enhances the m6A modification of rip3 mRNA mediated by the METTL3-METTL14 complex. This process increases the binding of YTHDF3 to rip3 mRNA, raising the protein levels of rip3 and thereby promoting SMC necroptosis, inflammatory responses, and contributing to the pathological development of AAA (Ji et al., 2023). Inhibition of METTL3/METTL14 has been shown to reduce SMC necroptosis, the inflammatory response, and the progression of AAA (Ji et al., 2023). Furthermore, Li et al. (2022) found that elevated METTL3 levels facilitate the maturation of primary microRNA-34a into miR-34a by targeting DGCR8 during apolipoprotein-induced AAA formation both *in vitro* and *in vivo*. The upregulation of miR-34a significantly inhibits sirtuin 1 expression, worsening the condition of AAA (Li et al., 2022). These findings underscore the crucial role of METTL3 in the development of AAA, suggesting it as a potential target for novel therapeutic interventions and as a diagnostic biomarker for this disease (Figure 2; Table 1).

### 2.4 METTL3 modulates HSC self-renewal and phenotypic transition

HSCs possess the distinctive capacity to balance self-renewal and phenotypic transition, which are pivotal in regulating angiogenesis and vascular functionality. Accumulating evidence underscores the vital role of METTL3 in orchestrating the self-renewal and hematopoietic reconstitution of HSCs. Notably, METTL3 knockout has been shown to increase HSC numbers in adult bone marrow while diminishing their self-renewal capacity. This phenomenon likely arises because METTL3 facilitates the expression of genes that maintain HSCs in a quiescent state, underscoring the indispensable role of METTL3 in adult bone marrow HSC self-renewal. Consistent with these findings, Lee et al. 39 demonstrated that METTL3 depletion in the adult hematopoietic system results in an expanded HSC pool within the bone marrow and a concomitant reduction in their reconstitution potential due to impaired differentiation. Specifically, METTL3 downregulation leads to insufficient Myc expression in HSCs, whereas Myc overexpression can rescue the differentiation defects induced by METTL3 deletion (Luo et al., 2022). Intriguingly, METTL3 silencing in myeloid cells does not produce a similar outcome, implying that METTL3-mediated m^6^A modification is essential for HSC differentiation regulation.

Moreover, Lv et al. (2018) reported that METTL3 methylates *Notch1* mRNA, thereby repressing Notch1 activity. YTHDF2 recognition of *Notch1* mRNA suggests that YTHDF2-mediated decay of *Notch1* mRNA is integral to the phenotypic transition of HSCs. Similarly, Zhang et al. (2017) indicated that METTL3 deletion in embryos inhibits HSC emergence by delaying YTHDF2-mediated mRNA decay of arterial endothelial genes *Notch1a* and *Rhoca*. Activation of Notch signaling in arterial endothelial cells of METTL3-deficient embryos obstructs the endothelial-to-hematopoietic transition, thus suppressing HSC generation. These observations highlight the critical role of METTL3-mediated m^6^A modification in dictating HSC fate during the endothelial-to-hematopoietic transition in vertebrate embryogenesis. Consistently, Cheng et al. (2019) found that METTL3 deletion in HSCs leads to ineffective symmetric differentiation, resulting in an expanded division of HSCs and an intermediate state resembling multipotent progenitors at both molecular and functional levels. Mechanistically, the abundance of *Myc* mRNA during HSC differentiation is regulated by METTL3-mediated m^6^A modification, establishing Myc as a marker of asymmetric and symmetric commitment in HSCs. METTL3 thus governs HSC identity and symmetric commitment, providing a general mechanism by which stem cells determine their fate during angiogenesis (Figure 2; Table 1).

In summary, METTL3 is crucial for maintaining the equilibrium between self-renewal and differentiation of HSCs during normal angiogenesis and vascular functions. Elevated METTL3 levels enhance self-renewal capabilities, whereas reduced levels promote the differentiation potential of HSCs.

### 2.5 The clinical and translational applications of METTL3 in vascular pathologies

Alterations in METTL3 expression or activity may indicate underlying pathological conditions and offer early signs of disease onset or progression. Hence, METTL3 holds potential as a biomarker for diagnosing and prognosticating vascular diseases. Consistently, we advocate for targeting METTL3 as a promising therapeutic strategy for vascular ailments. Considering METTL3’s crucial role in angiogenesis, maintaining a balanced METTL3 expression is essential for optimal angiogenesis. One way to achieve this balance is through targeted therapeutic interventions that modulate METTL3 expression or activity. For example, small molecule inhibitors or RNA-based therapies could be developed to downregulate METTL3 in scenarios where its overexpression leads to pathological angiogenesis, such as in cancer or AS. On the other hand, enhancing METTL3 activity could be advantageous in conditions marked by insufficient angiogenesis, such as ischemic heart disease or chronic wounds. Consequently, the development of specific inhibitors or enhancers of METTL3 activity could provide novel therapeutic approaches for vascular diseases. An integral part of developing METTL3-targeted therapies is the identification and validation of biomarkers that can predict patient response. Biomarkers associated with METTL3 expression, m^6^A modification levels, and downstream signaling pathways could help identify patients who would benefit most from these therapies. This approach aligns with precision medicine principles, where treatments are customized based on individual molecular profiles. High-throughput screening techniques and omics technologies, such as transcriptomics, proteomics, and metabolomics, could be utilized to discover and validate these biomarkers. Techniques like RNA sequencing and mass spectrometry could be employed to identify m^6^A-modified RNAs differentially expressed in diseased versus healthy vascular tissues.

To further investigate the therapeutic potential of METTL3 modulation, comprehensive preclinical and clinical studies are essential. These studies should evaluate the efficacy and safety of METTL3-targeted interventions in various models of angiogenesis-related diseases. For instance, using animal models of cancer, AS, and ischemic diseases can provide valuable insights into the *in vivo* effects of METTL3 inhibitors or enhancers. Additionally, it is important to assess the long-term impacts of such therapies, including potential side effects and resistance mechanisms that may arise with prolonged treatment. Moreover, precision medicine approaches that consider individual variations in METTL3 expression and activity could also be beneficial. Personalized therapies targeting METTL3 might be designed based on a patient’s genetic and epigenetic profile, ensuring optimal efficacy and minimal side effects. This would necessitate the integration of high-throughput sequencing technologies and bioinformatics tools to accurately assess METTL3-related alterations in patient samples. Another promising research area is the development of combination therapies that target METTL3 alongside other angiogenic regulators. Given the complexity of angiogenic signaling networks, modulating a single target is unlikely to achieve optimal therapeutic outcomes. Combining METTL3 inhibitors with other anti-angiogenic agents, such as VEGF inhibitors or immune checkpoint inhibitors, could enhance efficacy and overcome resistance mechanisms. Preclinical studies should evaluate the synergistic effects of such combinations and identify the most effective therapeutic regimens. Additionally, the potential off-target effects and toxicity of METTL3-targeted therapies must be thoroughly examined. As METTL3 is involved in various cellular processes beyond angiogenesis, its inhibition or enhancement could have unintended consequences. Comprehensive toxicological studies and careful monitoring during clinical trials are essential to ensure the safety of these therapies. Developing strategies to selectively target METTL3 in specific cell types or tissues could mitigate potential adverse effects.

## 3 Discussion

Various studies have shown that METTL3-mediated RNA m^6^A modification is integral to the regulation of gene expression in vascular component cells. This mechanism is crucial for maintaining vascular homeostasis and responding to pathological conditions. For example, METTL3 influences the differentiation and function of ECs by altering mRNA transcripts involved in angiogenesis. In VSMCs, METTL3-mediated m^6^A modification impacts the expression of genes related to cell proliferation and migration, which are key processes in atherosclerosis and restenosis development. Additionally, in HSCs, METTL3 affects fibrogenesis by regulating the stability of mRNA transcripts associated with ECM production. Despite these advancements, there is still a significant gap in understanding the exact molecular mechanisms by which METTL3-mediated m^6^A modification influences vascular biology. This review aims to bridge this gap by thoroughly interpreting the roles and mechanisms of METTL3 in various vascular events.

The analysis results of this review suggest that elevated levels of METTL3 are essential for maintaining the biological functions of ECs, VSMCs, and HSCs during angiogenesis, primarily by facilitating normal cell proliferation and differentiation to form adequate vessels. Under pathological conditions, however, METTL3 contributes to abnormal tube formation and angiogenesis by promoting excessive viability, proliferation, and migration of these cells, which ultimately accelerates the onset and progression of vascular diseases. Additionally, while METTL3 is crucial for regulating the biological functions of pericytes and ECs within vascular diseases, for example, low levels of METTL3 may help maintain their function and assist recovery from these conditions. However, studies for elucidating the relationship between METTL3 and pericytes in angiogenesis are still lacking, which needs further exploration. Moreover, despite the substantial advancements in elucidating the role of METTL3-mediated RNA m^6^A modification in vascular biology, there is a persistent need for extensive investigations to clarify its molecular mechanisms and therapeutic potential. Therefore, the critical role of METTL3 in both physiological and pathological angiogenesis underscores the necessity for finely-tuned regulatory mechanisms.

Exploring the downstream targets and signaling pathways influenced by METTL3 could provide deeper insights into its role in angiogenesis. It is crucial to identify the specific mRNA transcripts that are subject to METTL3-mediated m^6^A modifications and understand how these modifications alter their stability, translation, and function. This could reveal novel molecular targets for therapeutic intervention and enhance our understanding of the complex regulatory networks governing angiogenesis. Moreover, the interaction between METTL3 and various epigenetic regulators requires in-depth exploration. Angiogenesis is a complex process regulated by multiple layers, including DNA methylation, histone modifications, and non-coding RNAs. Understanding how METTL3-mediated m^6^A modifications interact with these epigenetic marks could uncover synergistic or antagonistic effects that intricately regulate angiogenesis. It is well understood that RNA molecules are subject to various modifications, such as pseudouridylation and 2′-O-methylation. The interplay between these modifications and m^6^A could unveil new levels of gene regulation crucial for vascular function. A particularly promising area of research is the relationship between METTL3 and other RNA modifications. For instance, the presence of multiple modifications on a single RNA molecule might form a complex code that finely adjusts the RNA’s translation, stability, and localization, thus influencing cellular responses to stress and injury.

Angiogenesis is not a uniform process; it varies significantly depending on the tissue context and the type of vascular bed involved. For instance, angiogenesis in the retina during diabetic retinopathy differs markedly from that in atherosclerotic plaques or tumor vasculature. Investigating the role of METTL3 in different tissue contexts and stages of development could provide a more comprehensive picture of its function. Additionally, understanding these nuances will enable the design of more specific and effective METTL3-targeted interventions. Therefore, the role of METTL3 in different cell types and tissues during angiogenesis must be dissected. To address this, future research should aim to elucidate the downstream targets of METTL3 in various vascular cells and determine how these targets contribute to disease phenotypes. Moreover, the role of the METTL3 complex in different stages of vascular disease, from initiation to progression and resolution, warrants detailed exploration. In early-stage AS, for instance, METTL3 might regulate inflammatory responses in ECs and monocytes, while in advanced disease, it could affect plaque stability by modulating VSMC behavior. Understanding these stage-specific roles could help in designing targeted interventions that are tailored to the disease stage. In addition to vascular diseases such as AS and restenosis, the role of METTL3 in other vascular conditions like hypertension, aneurysms, and vascular complications of diabetes should be investigated. Each of these conditions involves distinct pathological processes and cellular responses, and METTL3 is likely to play unique roles in each context. For instance, in hypertension, METTL3 might regulate the signaling pathways that control vascular tone and resistance, whereas in diabetic vasculopathy, it might influence the pathways involved in glucose metabolism and oxidative stress.

While current research has primarily focused on the role of METTL3 in angiogenesis, it is essential to consider the broader implications of METTL3 dysregulation in other physiological and pathological processes. Understanding how these processes intersect with angiogenesis could provide holistic insights into METTL3’s role in health and disease. For instance, in addition to its direct effects on ECs, VSMCs, and HSCs, METTL3 may also influence angiogenesis through its interaction with tumor surroundings. Tumors often hijack the angiogenic machinery to support their growth and metastasis, and METTL3 could be a pivotal player in this process. Understanding how METTL3 regulates the expression of pro-angiogenic factors, such as VEGF, HIF-1α, and angiopoietins, within the tumor microenvironment could provide new strategies for anti-angiogenic cancer therapies. In line with this, METTL3 has been implicated in various cellular processes, including stem cell renewal, immune response, and neural development. The interplay between METTL3 and the immune system also deserves attention. Angiogenesis and immune responses are closely linked, particularly in the context of cancer and chronic inflammation. METTL3’s role in modulating immune cell function and its impact on the tumor microenvironment could uncover new avenues for immunotherapy. Investigating how METTL3 influences immune cell infiltration, activation, and cytokine production in angiogenic contexts could lead to novel combination strategies that harness both anti-angiogenic and immunomodulatory effects. Moreover, advancing our understanding of METTL3’s role in developmental angiogenesis could provide insights into congenital vascular disorders and potential therapeutic approaches for these conditions. Studying METTL3 function in embryonic and neonatal models could reveal critical windows of development where METTL3 activity is essential, offering opportunities for early intervention in cases of developmental vascular anomalies.

In summary, this review underscores the importance of maintaining a precise equilibrium in METTL3 expression for optimal angiogenesis. Future research should prioritize the development of targeted therapies, the dissection of downstream targets and signaling pathways, the exploration of interactions with other epigenetic modulators, and the consideration of precision medicine strategies. Moreover, therapeutic targeting of METTL3 offers a multifaceted approach to modulating angiogenesis across various pathologies. Future studies should emphasize thorough preclinical and clinical evaluations, the identification of predictive biomarkers, and the formulation of combination therapies. By addressing these areas, we can better leverage METTL3’s therapeutic potential and deepen our understanding of its complex role in angiogenesis and beyond. Collectively, this review delivers an in-depth analysis of METTL3’s mechanisms in vascular biology and evaluates its clinical applications, highlighting current research limitations and future directions, while proposing strategies to overcome these challenges, thus serving as a valuable reference for scholars in the field.
